# Sales of antidepressants, suicides and hospital admissions for depression in Veneto Region, Italy, from 2000 to 2005: an ecological study

**DOI:** 10.1186/1744-859X-10-24

**Published:** 2011-09-30

**Authors:** Giuseppe Guaiana, Margherita Andretta, Eric Griez, Bruno Biancosino, Luigi Grassi

**Affiliations:** 1 Department of Psychiatry, University of Western Ontario, Regional Mental Health Care Saint Thomas, Saint Thomas, Ontario, Canada; 2 Servizio Farmaceutico ULSS 20, Via Valverde 42, 37122 Verona, Italy; 3 University of Maastricht, Department of Psychiatry and Neuropsychology, Vijverdalseweg 1 Maastricht, The Netherlands; 4 Azienda USL Ferrara, Dipartimento di Salute Mentale e Dipendenze Patologiche, Via Ghiara 38, 44100 Ferrara, Italy; 5 University of Ferrara, Department of Medical-Surgical Disciplines of Communication and Behavior, Section of Psychiatry, Corso Giovecca 203, 44100 Ferrara, Italy

## Abstract

**Background:**

Increased prescription of antidepressants has been consistently associated with a decrease in suicide rates in several countries. The aim of this study is to explore antidepressant consumption, suicide rates and admission for depression in the Veneto Region, Italy, in order to see whether the same pattern could be detected.

**Methods:**

Data from the Italian Ministry of Health (admissions for depression), the Pharmacy Service of a Local Health Unit (antidepressant prescribing) and from the Epidemiological System of the Veneto region (suicide rates) were collected from 2000 to 2005 for the Veneto region.

**Results:**

Suicide rates did not show any marked increase but were stable in males and females. Antidepressant prescribing increased exponentially over the period examined, whilst admissions for depression markedly decreased. The trend for an exponential increase in antidepressant prescribing in the Veneto region is shared with other countries and locales.

**Conclusions:**

It is possible that the increase in antidepressant prescribing might be associated with earlier treatment of depression, thus decreasing the likelihood of aggravation of depression.

## Background

Antidepressant prescribing has risen in several countries worldwide over the last 20 years, mainly after the introduction of selective serotonin reuptake inhibitors (SSRIs) [1,2]. This increase may be the result of better treatment and recognition of depression [3]. However, some concerns have been raised over the fact that antidepressant use may increase the risk of suicide [4]. Ecological studies have shown some evidence that more widespread antidepressant use corresponds to a decrease in suicide rates [1], although this finding is disputed [5]. Some studies show that treating more depressed patients with antidepressants may prevent suicides [6]. Moreover, a meta-analysis by Barbui and colleagues [7] on observational studies, showed that use of SSRIs is associated with a reduced risk of suicide in adults with depression, particularly in people aged 65 or over.

Other than suicide and antidepressant prescribing, the pattern of hospital admissions for depression can be another valid indicator when exploring trends in the prevalence of depression and may shed some light on the relationship between suicide rates and depression. In one of the few ecological studies on the topic, Vyssoki and colleagues found that increased hospital admissions for depression were linked to a decrease in suicide rates in Austria between 1989 and 2008 [8]. These findings are in line with those of Roy [9], who found that patients who commit suicide at their first attempt had had fewer admissions for depression. It appears that hospital admissions may have a protective effect on suicide rates. Hospital admissions rates can also be considered a proxy for depression severity [10,11] and can offer some insight on the trend of severely depressed people over time.

Evidence shows that depression is a major cause of suicide [12], and that severe depression is associated with higher suicide risk [13]. The time trend of hospital admissions for depression has been investigated in several studies [2,14,15]. Shajan and colleagues [15] found that admissions for depression declined in females and increased in males between 1980 and 1995 in Scotland; however, Walsh [14] found that there was no change in admissions in males in Ireland between 1975 and 1995. Guaiana and colleagues [2] found that admissions in Italy did not decline between 1986 and 1998. Analysing combined trends for hospital admissions for depression, suicide and antidepressant prescribing can offer some insight on the impact of antidepressant prescribing on admissions for depression and suicide rates. This analysis has been carried out for some European countries [2,5]. However, only few studies have focused on selected regions of a country. Italy is a diverse country with some notable differences between the North and the South [16]. Consequently, trends in the whole of Italy may not be representative of a specific region.

Veneto is a north-east Italian region with a population of about 5 million people [16]. It has an average yearly numbers of suicides of 327 [17]. Half of suicides occur in people aged 52 or older [17]. Males account for three-quarters of suicides [17]. The most commonly used method is hanging [17].

The aim of this study was to examine the trend in admissions for depression, antidepressant prescribing and suicide in this large Italian region, with a view to investigating how the pattern of antidepressant sale is related to suicide and admissions for depression, considered a proxy for depression severity.

## Methods

Data on admissions for depression were collected by the Italian Health Ministry internet database on Hospital discharges (Scheda di Dimissione Ospedaliera (SDO)). The official database is freely available on the internet [18], and includes data from 2000 to 2005. For every patient discharged from an Italian hospital, an official discharge form (the SDO) needs to be completed and sent to the Ministry of Health. The SDO includes demographic data (age, sex, region of discharge) and International Classification of Diseases, Ninth Revision, Clinical Modification (ICD-9-CM) discharge diagnosis. Data were selected for the Veneto region and for the following ICD-9-CM diagnostic codes: 296.20 to 295.26 (major depression single episode), 296.30 to 296.36 (major depression recurrent episode), 296.82 (atypical depressive syndrome), 298.0 (depressive psychosis), 300.4 (neurotic depression), 311 (depressive disorder, not otherwise classified). Data on age were grouped in the following age bands: 0-14 years of age, 15-64, and 65 and over. There is no specific validation study of the SDO. However, the Italian Ministry of Health runs its own validation audit and it publishes the results in their annual report [19]. A raw rate of admission per 100,000 population was calculated for each age band and sex, for each year, as well as an age-standardised total rate of admissions for depression per 100,000 population, for each year and sex. The standard population is the 2011 Census population.

We also collected data on psychiatric bed availability in Veneto Region from 2000 to 2005, from the ISTAT statistical yearbook [16].

Data on antidepressant (AD) prescriptions were collected by the pharmacy service of one of the Local Health Units (LHUs) in Veneto, from 2000 to 2005. No data is available after 2005. Data were available for 12 out of 21 LHUs. The population of 12 LHUs refers to all inhabitants of the LHU area with no further restrictions. The distribution of classes of ages and gender is almost the same for the 12 LHUs and the Veneto region as a whole (percentage of children aged 0-14 years: 13.8% vs 13.9%, percentage of older people over 65 years: 19.8% vs 19.5%, percentage of females: 51.2 vs 51.1). Furthermore, prescriptions of antidepressants in the 12 LHUs are very similar to that of the Veneto region (in 2005 then number of boxes of antidepressants prescribed were 0.46 vs 0.44). Based on these assumptions, the sample of 12 LHUs can be considered as representative of the whole Veneto region. For each AD, the number of packages sold was converted into defined daily doses (DDDs) per 1,000 inhabitants per day (DDD/1,000/day). The DDD is the international unit of drug utilisation approved by the World Health Organization for drug use studies [20]. The DDD is a theoretical unit of measurement defined as the assumed average maintenance daily dose for a drug, used for its main indication in adults. The DDD/1,000/day indicates how many people per 1,000 of the population have in theory received a standard dose (that is, the DDD) of a particular medication or category of medication daily.

Data on numbers of suicide deaths according to the Ninth Revision of the Italian International Classification of Diseases (ICD-IX) in each sex and age group and estimates of the resident population in Veneto were collected from the regional epidemiological system (Sistema Epidemiologico Regione Veneto (SER)), which is the local government-funded regional statistical office, from 2000 to 2005. Data were age standardised by the SER for both sexes.

A statistical model using ordinary least squares linear regression was employed, based on the model developed by Preti and Miotto [21]. The model was used in the present study to test for changes over time in suicide rates and admission rates for depression. Rates were the dependent variable and years were the independent variable. Linear regression analysis of rates over 6 years (2000 to 2005) was performed. A two-tailed t test was also performed to test for the hypothesis of a significant slope.

A Spearman ρ correlation coefficient per each sex and age strata across the time period examined (2000-2005) was calculated for AD DDD and admissions for depression rates. We also calculated a Spearman ρ correlation coefficient for AD DDD and suicides rates for each sex, between 2000 and 2005 and a Spearman ρ correlation coefficient for admissions for depression and suicide rate, for each sex, between 2000 and 2005. SPSS for Windows V. 19 (SPSS, Chicago, IL, USA) was used to perform all the calculations.

## Results

### Hospital admissions for depression and psychiatric beds

We performed a separate analysis of admissions for each age band and sex for the years 2000 to 2005. The pattern of admission for the age band 0-14 males was quite erratic, with a drop in 2001 and a steady increase from 2002 to 2005. Females aged 0 to 14 also showed an erratic pattern with a decline in admissions from 2000 to 2001, followed by a drop in 2002, a peak in 2004 and another decline in 2005. It is very likely that this erratic pattern reflects the small numbers of admissions. The linear regression model showed no significant trend both for males (slope -0.036, 95% CI -0.312 to 0.384, SE 0.125, adjusted R^2 ^-0.225, F 0.082, *P *= 0.788) and for females (slope -0.069, 95% CI -0.544 to 0.406, SE 0.171, adjusted R^2 ^0.201, F 0.162, *P *= 0.708).

The 15-64 age band showed, instead, a consistent trend toward reduction in admission between 2000 and 2005, for males (slope -1.465, 95% CI -1.837 to -1.093, SE 0.134, adjusted R^2 ^0.960, F 119.642, *P *= 0.0001) and, to a higher degree, for females (slope -6.379, 95% CI -7.291 to -5.466, SE 0.329, adjusted R^2 ^0.987, F 376.559, *P *= 0.0001). People aged 65 or older also showed a downward trend. Admissions for males over 65 increased from 2000 to 2001 and then steadily decreased (slope -8.994, 95% CI -15.247 to -2.741, SE 2.252, adjusted R^2 ^0.749, F 15.948, *P *= 0.016). Female admissions showed a decreasing trend from 2000 to 2005 (slope -22.508, 95% CI -30.518 to -14.497, SE 2.885, adjusted R^2 ^0.923, F 60.861, *P *= 0.001).

The total age-standardised admission rates for depression for each sex are shown in Table 1. Overall, the admissions for depression declined both in males and in females from 2000 to 2005. The number of beds in the Veneto region changed from a maximum of 796 in 2000 to a minimum of 733 in 2003. The number of beds rose again up to 763 in 2004 and 757 in 2005.

**Table 1:** Age-standardised admission rate per 100,000 population for depression for each sex, 2000-2005

	Males	Females
2000	20.92	46.52
2001	21.42	44.96
2002	20.94	42.26
2003	20.51	38.94
2004	20.33	37.51
2005	19.54	35.13

### Antidepressant drugs prescribing

**Figure 1:**
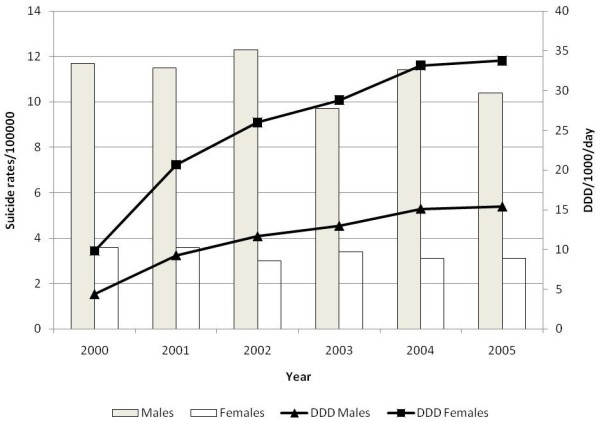
**Suicide rates (bars) and antidepressant prescribing (line) among males and females in Veneto from 2000 to 2005**.

The analysis shows that AD consumption in the Veneto region increased exponentially from 2000 to 2005, both for males and for females (see Figure 1).

If the data is broken down by age (see Table 2), there is an increased use of antidepressants on moving to the higher age bands. This finding is consistent among males and females for all the years examined. AD prescribing is at least double that in males and females over 65 compared to people aged 15-64 years. Looking at the prescribing trend, we find that in the 0-14 age band AD consumption steadily increased between 2000 and 2005, both in males and in females. Looking at the 15-64 age band, we find that AD prescription increased exponentially from 2000 to 2005 with an almost identical trend in males and females. Males, however, were prescribed less DDD than females. The 65 and over age band shows the same trend as the 15-64 year olds, with an exponential increase in AD DDD with almost identical trends for males and females, and males prescribed less DDD than females.

**Table 2:** Defined daily doses/1,000/day for antidepressants (ADs) divided by age and sex

	Males			Females		
	
	0-14	15-64	65+	0-14	15-64	65+
2000	0.05	3.63	12.51	0.02	7.60	22.73
2001	0.11	8.76	20.85	0.11	18.56	39.75
2002	0.17	11.05	25.67	0.18	23.58	48.99
2003	0.22	12.34	28.06	0.28	25.99	54.39
2004	0.31	14.60	31.35	0.4	30.43	60.94
2005	0.35	15.12	30.61	0.53	31.73	59.62

### Suicides

Suicide rates per 100,000 population in Veneto showed some minor changes in males over the period examined, with a maximum of 12.3 in 2002 and a minimum of 9.7 in 2003. The rate of suicide did not change markedly in females, oscillating between a maximum of 3.6/100,000 in 2000 and 2001 and 3.1/100,000 in 2004 and 2005 (see Figure 1). The linear regression model did not yield a statistically significant result, both for males (slope -0.269, 95% CI -0.863 to 0.326, SE 0.214, adjusted R^2 ^0.103, F 1.572, *P *= 0.278) or for females (slope -0.103, 95% CI -0.242 to 0.036, SE 0.05, adjusted R^2 ^0.393, F 4.235, *P *= 0.109).

### Correlation

The Spearman ρ correlation coefficients for AD DDD and admission rates for depression are shown in Table 3.

**Table 3:** Spearman ρ correlation coefficients and *P *values for antidepressant defined daily doses (AD DDD) and admissions rates for depression

Admissions for depression and DDD	Spearman ρ/*P *value
Males aged 0-14	-0.08 (NS)
Females aged 0-14	-0.3 (NS)
Males aged 15-64	-1 (*P *< 0.01)
Females aged 15-64	-1 (*P *< 0.01)
Males aged 65+	-0.94 (*P *< 0.05)
Females aged 65+	-0.89 (*P *< 0.05)

NS = not significant.

It appears that there is a high inverse correlation between antidepressant prescribing and admissions for depression for the age strata 15-64 and 65+, both in males and in females. No significant correlation was found between antidepressant prescribing and admissions for depression in the age strata 0-14, for either sex.

The Spearman ρ correlation coefficients for AD DDD and age-standardised suicide rates are shown in Table 4. There is no correlation between suicide rates and AD DDD between 2000 and 2005, for either sex.

**Table 4:** Spearman ρ correlation coefficients and *P *values for antidepressant defined daily doses (AD DDD) and suicide rates

AD DDD and suicide rates	Spearman ρ/*P *value
Males	-0.66 (NS)
Females	-0.61 (NS)

NS = not significant.

The Spearman ρ correlation coefficients for total admissions for depression and age-standardised suicide rates for each sex are shown in Table 5. There is no correlation between suicide rates and admissions for depression between 2000 and 2005, for either sex.

**Table 5:** Spearman ρ correlation coefficients for admissions for depression and suicide rates for each sex

Total admissions and suicide rates	Spearman ρ/*P *value
Males	0.66 (NS)
Females	0.62 (NS)

NS = not significant.

## Discussion

The present study aimed to assess trends in hospital admissions for depression, considered as a proxy for depression severity, and to relate these trends with antidepressant prescription and suicide rates in the region of Veneto between 2000 and 2005. Additionally, suicide rates were analysed in order to assess a possible link between antidepressant use and suicide. Essentially, we found a marked decline in admissions for depression, with no major change in psychiatric bed availability, an exponential increase in antidepressant prescribing, and no change in suicide rates. Also, the results show that the trend of suicide rates does not correlate with the changes in admission rates for depression. It also appears that the increase in AD prescribing does not correlate with changes in suicide rates. It may be possible that the relationship between AD prescribing and suicide rates may work only for some age strata. Older people use antidepressants far more than younger people. This finding has been confirmed for the whole of Italy [22]. Unfortunately, we have no information on suicide rates divided by age. It appears that antidepressants are very effective in preventing suicide among older people [7]. We also have no data on the proportion of SSRIs that make up the total number of ADs prescribed. However, we can say that it is likely that the majority of antidepressants prescribed in Veneto are SSRIs and newer ADs. The study by Guaiana and colleagues [2] showed that SSRIs and newer ADs had an exponential increase in prescription, whilst the prescription of older tricyclic ADs did not change. Also, two other recent studies performed in different Italian regions [23,24] showed that the prevalence of SSRI use had markedly increased. The first and most striking finding is the sharp decline of hospital admissions for depression. This decrease affected both the age strata 16-64 and 65+ age band, as well males and females. The data relating to the 0-14 age strata are not reliable due to the small numbers involved. This finding is at odds with that of Vyssoki and colleagues [8], who found that an increase in hospital admissions for depression was in parallel with a decrease in suicide rates. There are several possible explanations to the findings in the 16-64 and 65+ age bands. In theory, fewer hospital admissions may just reflect a decrease in the general prevalence of depression, or a decrease in recognition of depression, or both. There is no evidence to suggest either. On the contrary, worldwide data have repeatedly documented an increase in the incidence and/or awareness of depression both amongst the general population and medical practitioners [5]. Moreover, both trends would be at odds with our second finding of an impressive increase in antidepressant prescription in the same region at the same time. Another possibility is that the observed decrease in hospital admission merely reflects a decrease in bed availability. This however does not seem to be the case as the number of beds did not decline. The most likely explanation of the observed decline in hospital admissions for depression between 2000 and 2005 is that depression became better diagnosed, and therefore better treated. This notion becomes particularly salient when considering our next finding of an approximately fivefold increase in antidepressant use in the same period. It is most plausible that the observed increase in antidepressant prescribing reflects a better effort in recognising depression, and therefore earlier and more effective treatment. The benefits of early intervention have been widely documented [25], resulting in the shortening of the depressive episode. If, as mentioned above, hospital admissions are a valid indicator of depression severity [10,11], then it is arguable that the decrease in hospital admissions is directly linked to an increase in effective prescription of antidepressants. It is of note that a worldwide increase in the prescription of antidepressants has been observed over the last 10-15 years [5].

There have been some reports showing that admissions for depression increased or did not change [2,14,15]. Shajahan [15] reported that admissions increased in males in Scotland. They hypothesised that this may be due to increased recognition of depression or an increase in the health-seeking behaviour of males. The study by Shajahan refers to an earlier timeframe (1980-1995). It is possible to hypothesise that since the late 1990s, there has been an increased awareness of depression, possibly leading to an increase in the number of the people treated with antidepressants, which eventually led to an overall decrease in depression severity and therefore in admissions for depression. The increased awareness for depression may likely have happened in primary care scenarios. It appears that general practitioners prescribe more and more ADs in some European regions [8]. To corroborate this hypothesis, Munoz-Arroyo and colleagues [26] found that prescription of antidepressants by general practitioners doubled in Scotland from 1992 to 2000. Also, the studies by Walsh [14] and Guaiana *et al*. [2], examining admissions in Ireland and in Italy, respectively, referred to an earlier timeframe. Our hypothesis is that there has been a time lag between increased prescription of antidepressants and reductions in admissions for depression. In Italy, antidepressant prescribing has increased exponentially only since 1995 [2]. We hypothesise that it may have taken some years to see an effect on depression and admissions for depression.

Also, it is of interest to note that the decrease in admissions for depression is not associated with change in suicide rate, as we found no correlation between admissions for depression and suicide rates, for both sexes. Our final finding is that overall suicide rates remained unchanged during the period under examination, in spite of the observed exponential increase in use of antidepressants. If antidepressants were associated with an increase in suicide rates, as pointed out by Healy and colleagues [4], there would have been an increase in suicide rates, which did not happen. Vichi and colleagues examined suicide rates in Italy between 1980 and 2002 [27]. They concluded that the decline in suicide rates was possibly a consequence of the decrease in the incidence of mental disorders as a result of the development of an integrated and community-based mental health system, which in turn may have led to decreased suicide rates as a consequence of early detection of mental disorders, including depression. AD prescribing may be part of this picture, as earlier detection of mental disorders may have led to an increase in AD prescribing. Our findings are more in line with the data examined by Isaacson and colleagues [5,6], and Barbui and colleagues [7] where suicide rates showed an opposite trend to antidepressant prescribing. Also, Khan and colleagues [28] showed that SSRIs do not induce suicide more than other ADs or even placebo. Regardless, our study failed to find any argument suggesting that an increase in antidepressant use is associated with increased risk of suicide.

The present study suffers from some limitations, however. First we assumed that all antidepressants are prescribed and used for depression. This is not entirely true, as antidepressants are increasingly used for anxiety disorders as well, and this is the case in Italy [29]. Anxiety disorders are at least as prevalent as depression. Both types of disorders are highly comorbid. Our database does not make it possible to find out the specific disease that required the prescription of the antidepressant. However, since anxiety and depression are closely intermingled, the former often being an indicator of the severity of the latter and vice versa [30], this limitation does not necessarily invalidate our main conclusions. Secondly, data on antidepressant prescribing does not cover the entire Veneto region, but only some of the LHUs. We have assumed that the data can be extended to the whole region but we cannot rule out a possible bias in data collection. Also, the study covers a relatively limited period of time; only 6 years. Nevertheless, clear trends can be seen. Thirdly, we cannot exclude the possibility that that the downward trend in hospital admissions for depression in Veneto and in Italy could be part of a broader picture, where admissions for mental health problems as a whole are decreasing. Moreover, the DDD only reflects prescription and not current usage. It is not possible to take for granted that all people who receive a prescription will use it. It must also be said that the reliability of data on admissions, suicides and DDD may be questioned, as happens for any large-scale data collection. Another point to consider is that suicide is not only linked to depression but also to schizophrenia, bipolar disorder and substance misuse [31-33]. Our data do not include admissions for those conditions. Findings may have been different if we had included admissions for those mental disorders. Finally, the naturalistic design of this analysis is by definition subject to ecological fallacy, and therefore no causal relationships can be definitely established.

## Conclusions

In spite of the limitations outlined above, we conclude that the marked increase observed in prescription of antidepressants has been attended by a striking decrease in hospital admissions for depression in the same period. The exponential change in antidepressant use was not accompanied by any relevant change in suicide rates. Further studies will be required to refute or corroborate these findings.

## Competing interests

The authors declare that they have no competing interests.

## Authors' contributions

GG and MA participated in the design of the study. GG, BB, LG and EG devised the statistical model and contributed to the Discussion section.
